# The Human Gut Microbiome – A Potential Controller of Wellness and Disease

**DOI:** 10.3389/fmicb.2018.01835

**Published:** 2018-08-14

**Authors:** Zhi Y. Kho, Sunil K. Lal

**Affiliations:** School of Science, Tropical Medicine and Biology Platform, Monash University, Subang Jaya, Malaysia

**Keywords:** microbiome, colorectal cancer, dysbiosis, celiac disease, obesity, autism, inflammatory bowel disease, *Clostridium difficile* infection

## Abstract

Interest toward the human microbiome, particularly gut microbiome has flourished in recent decades owing to the rapidly advancing sequence-based screening and humanized gnotobiotic model in interrogating the dynamic operations of commensal microbiota. Although this field is still at a very preliminary stage, whereby the functional properties of the complex gut microbiome remain less understood, several promising findings have been documented and exhibit great potential toward revolutionizing disease etiology and medical treatments. In this review, the interactions between gut microbiota and the host have been focused on, to provide an overview of the role of gut microbiota and their unique metabolites in conferring host protection against invading pathogen, regulation of diverse host physiological functions including metabolism, development and homeostasis of immunity and the nervous system. We elaborate on how gut microbial imbalance (dysbiosis) may lead to dysfunction of host machineries, thereby contributing to pathogenesis and/or progression toward a broad spectrum of diseases. Some of the most notable diseases namely *Clostridium difficile* infection (infectious disease), inflammatory bowel disease (intestinal immune-mediated disease), celiac disease (multisystemic autoimmune disorder), obesity (metabolic disease), colorectal cancer, and autism spectrum disorder (neuropsychiatric disorder) have been discussed and delineated along with recent findings. Novel therapies derived from microbiome studies such as fecal microbiota transplantation, probiotic and prebiotics to target associated diseases have been reviewed to introduce the idea of how certain disease symptoms can be ameliorated through dysbiosis correction, thus revealing a new scientific approach toward disease treatment. Toward the end of this review, several research gaps and limitations have been described along with suggested future studies to overcome the current research lacunae. Despite the ongoing debate on whether gut microbiome plays a role in the above-mentioned diseases, we have in this review, gathered evidence showing a potentially far more complex link beyond the unidirectional cause-and-effect relationship between them.

## Introduction

The human microbiome comprises of collective genomes of microbiota inhabiting us, namely protozoa, archaea, eukaryotes, viruses and predominantly bacteria that live symbiotically on and within various sites of the human body. Examples of occupied habitats include our oral cavity, genital organs, respiratory tract, skin and gastrointestinal system (Lloyd-Price et al., 2016). The human microbiota is estimated to be ∼10^13^–10^14^ microbial cells, with around 1:1 microbial cells to human cells ratio (Sender et al., 2016). These numbers are derived from the total bacterial cells in colon (3.8 × 10^13^ bacteria), the organ that harbors the densest number of microbes (Sender et al., 2016). The diverse gastrointestinal microbiota is predominantly composed of bacteria from three major phyla, namely *Firmicutes, Bacteroidetes*, and *Actinobacteria* (Tap et al., 2009). This diverse and complex microbiome serves as a functional expansion of host genomes, and is estimated to harbor 50- to 100-fold more genes, compared to the host. These extra genes have added various types of enzymatic proteins which were non-encoded by the host, and play a critical role in facilitating host metabolism, thus contributing to the regulation of host physiology (Hooper and Gordon, 2001). Until recent decades, the properties of the human microbiome and the host–microbiota interactions have been largely unknown due to technology limitations especially in examining non-cultivable microbes of interest, and lack of population-scale data depicting the microbiota compositions and functions. However, advances in sequencing technologies and subsequent large-scale sequence-based microbiome projects such as the Human Microbiome Project (HMP) consortium funded by The United States National Institutes of Health (NIH), as well as the MetaHIT (Metagenomics of the Human Intestinal Tract) consortium funded by the European Commission, have served as catalysts in nourishing research on the human microbiome. These large-scale endeavors both share similar missions in characterizing the human microbiome and their roles in health and disease states, with MetaHIT solely focusing on gut microbiome. Several analyses have been incorporated in these meta-omics projects including 16S ribosomal RNA (rRNA) sequencing to taxonomically characterize the microbiota communities; Whole Genome Shotgun (WGS) metagenomic sequencing of body-site specific whole community DNA, followed by reference genome mapping, metagenomic assembly, gene cataloging and metabolic reconstruction, to facilitate maximal capture of organismal and functional data of human microbiota (The Human Microbiome Project Consortium, 2012).

Due to the inherent complexity and heterogeneity of the human microbiome, experiments are required to counteract the limitation of empirical methods in examining the causation or correlation links between microbiota disequilibrium (dysbiosis) and human diseases. Robust experimental modeling enables systematic manipulation of variables to investigate hypotheses deduced from “omics” studies. For this, the application of ‘humanized’ gnotobiotic animal model that harbors defined collection of sequenced microbial communities, has gained momentum in recent years in microbiome research (Faith et al., 2010). This allows proof-of-mechanism study to examine the potential impacts of diet (Turnbaugh et al., 2009b), antibiotic, environmental toxicants (Stedtfeld et al., 2017) and host genotypic variation (Ley et al., 2005) on the microbiota and disease manifestation, due to changes in microbiota composition, transcriptomes, proteomes or metabolomes post-induced variations can be extracted and characterized to understand the operation of the microbiota. Besides, ‘humanized’ gnotobiotic mice can be used in preliminary testing of the therapeutic efficacy in treating dysbiosis-associated diseases, as they allow monitoring of the pharmacokinetic–pharmacodynamic changes in microbial communities, thus facilitating optimization of treatment and dosage regime (Mahe et al., 1987; Silva et al., 1999).

Undoubtedly, these efforts shed light on the clinical significance of the human microbiome which is pretty much a ‘black box.’ Although the human microbiome research is still at its preliminary stage, the findings are deemed intriguing yet promising in terms of filling the knowledge gap in microbiome-host relationships, and their role in disease pathogenesis, as well as therapeutic value, which requires more in-depth investigations to uncover this exciting yet mysterious field of research. Below, we review recent investigations specifically related to the bacterial microbiome in the GIT – the largest microbial reservoir of the human body. Gut microbiota and the microbial-synthesized metabolites are discussed along with their roles in human wellness and normal functioning. Further, we review several studies related to the gut microbiome dysbiosis and its association with specific human diseases. We introduce novel microbiome-based therapy employed in specific disease conditions to ‘restore’ wellness and ameliorating dysbiosis-associated diseases. Finally, we discuss future directions and research areas that require further elucidation in order to better understand the human microbiome and its relationship with the host.

## The Gut Microbiome and Its Multifarious Functions

The symbiotic relationship between the gut microbiota and the host is regulated and stabilized by a complex network of interactions that encompass metabolic, immune, and neuroendocrine crosstalk between them. This crosstalk is potentially mediated by microbial-synthesized metabolites which exhibit pleiotropic effects, including acting as signaling molecules in regulating host neuro-immune-inflammatory axes that could physiologically link gut with other organ systems. The predominant functions of gut microbiota and the associated key metabolites in governing host wellness are depicted in the following subsections, with some other microbial metabolites being described in **Table 1**.

**Table 1:** Metabolites contributed by gut microbiota and their respective functions.

Metabolites	Functions	References
Short-chain fatty acids (SCFAs):	Regulate host metabolic pathways via G-protein-coupled receptor GPR41 or GPR43 -mediated signaling:	Blottière et al., 2003; Backhed et al., 2004; Xiong et al., 2004; Samuel et al., 2008; Tolhurst et al., 2012; De Vadder et al., 2014
E.g., Acetate, butyrate, propionate, hexanoate, valerate	energy homeostasis; synthesis of glucagon-like peptide 1 (GLP-1); increase leptin production. Improve glucose tolerance and insulin sensitivity. Potent histone deacetylase (HDAC) inhibitor - regulation of intestinal cell proliferation. Intestinal gluconeogenesis, lipogenesis, suppression of fasting-induced adipose factor Fiaf (lipoprotein lipase inhibitor) in intestinal epithelium. Immunomodulatory effect, activate dendritic cells, gut immunity.	
Indole derivatives:	IPA as powerful antioxidant, inhibitor of amyloid-beta fibril formation, and exhibits neuroprotective and cytoprotective effects against a variety of oxidotoxins.	Bendheim et al., 2002; Deguchi et al., 2002; Wikoff et al., 2009; Venkatesh et al., 2014
E.g., Indole, indoxyl sulfate, indole-3-propionic acid (IPA)	IPA regulates intestinal barrier function via the xenobiotic sensor, pregnane X receptor (PXR), in which it reduces intestinal inflammation (downregulates enterocyte pro-inflammatory cytokines TNF-α), and regulate intestinal permeability and mucosal integrity (upregulates junctional protein-coding mRNAs). Indoxyl sulfate as uremic toxin that accumulates in the blood of patients with impaired excretion system.	
Bile acid metabolites: E.g., Deoxycholic acid (DCA), lithocholic acid (LCA)	Activate host nuclear receptors and cell signaling pathways: regulation of bile acid, cholesterol, glucose, lipid, and energy metabolism. Exhibit antimicrobial effects.	Inagaki et al., 2006; Hylemon et al., 2009
Choline metabolites:	Modulate lipid metabolism and glucose homeostasis.	Dumas et al., 2006; Wang Z. et al., 2011
E.g., Choline, trimethylamine N-oxide (TMAO) and betaine	Contribute to non-alcoholic fatty liver disease and cardiovascular disease.	
Phenolic derivatives:	Antimicrobial effects: repress pathogenic microbes, influence gut microbiota composition, maintenance of intestinal health.	Larrosa et al., 2006, 2010; Lee H.C. et al., 2006; Selma et al., 2009
E.g., 4-OH phenylacetic acid, equol, urolithins, enterolactone, enterodiol, 8-prenylnaringenin, 2-(3,4-dihydroxyphenyl)acetic acid, 3-(4-hydroxyphenyl)propionic acid, 5-(3,4-dihydroxyphenyl)valeric acid	Protective effect against oxidative stress. Estrogen-modulating effect. Platelet aggregation inhibition effect. Urolithin exhibits anti-inflammatory and cancer chemopreventive effects.	
Vitamins:	Energy production, red blood cell formation, as enzymatic cofactor for diverse biochemical reactions.	LeBlanc et al., 2011; Nicholson et al., 2012; Forster et al., 2017; Lerner et al., 2017b
E.g., Thiamine (B1), riboflavin (B2), niacin (B3), pyridoxine (B6), pantothenic acid (B5), biotin (B7), folate (B11–B9), cobalamin (B12), and menaquinone (K2)	DNA replication, repair and methylation, regulating cell proliferation. Production of nucleotides, vitamins and amino acids. Enhance immune functioning.	
Polyamines:	Sustain high proliferation rate of Intestinal epithelial cells.	Guo et al., 2003, 2005; Perez-Cano et al., 2010; Johnson et al., 2015; Rooks and Garrett, 2016
E.g., putrescine, spermidine, and spermine	Dysregulated polyamine metabolism possibly enhances cancer development. Enhance intestinal barrier integrity and function via stimulating synthesis of intercellular junction proteins [occludin, zonula occludens-1 (ZO-1), E-cadherin]. Enhance maturation of intestinal and systemic adaptive immune system. Spermine inhibits pro-inflammatory M1 macrophage activation.	

### Metabolism

Human fecal sample analysis using 16S ribosomal RNA and metagenomic sequencing techniques reveal significant enrichment in metabolism of polysaccharides, amino acids, xenobiotics and micronutrients conferred by gut microbiota, suggest that these indigenous microbes facilitate host energy harvesting and metabolic efficiency (Gill et al., 2006). These findings were further validated by germ-free (GF) mice experiments where it was found that germ-free mice had 40% lower epididymal fat and an additional 10–30% food consumption was needed to maintain the same body mass as mice with normal microbiota (Backhed et al., 2004). Gut microbiota is important in fermenting unabsorbed starch and soluble dietary fiber. The fermented end products exist in the form of a SCFAs. SCFA (such as butyrate, propionate, acetate and pentanoate) act as one of the energy substrates for the host (Salminen et al., 1998) thereby contributing an extra 10% daily dietary energy for utilization by the host for other metabolic processes (Payne et al., 2012). Microbial-synthesized SCFAs contribute 70% of ATP production in colon, with butyrate as the preferred fuel for colonocytes (Firmansyah et al., 1989; Donohoe et al., 2011). Butyrate-producing microbes rescued the deficit mitochondrial respiration, ATP synthesis and autophagy in colonocytes of germ-free mice (Donohoe et al., 2011), proving the importance of butyrate in colonic cellular respiration and energy production. Furthermore, SCFAs which are the ligands for G protein-coupled receptor 41 (GPR41) expressed by a subset of the gut epithelial enteroendocrine cells, had been shown to regulate energy homeostasis by stimulating GPR41-mediated leptin production in mouse adipocytes, in which this multifunctional circulating hormone, leptin exhibits pleiotropic effects on a vast range of host physiological functions such as energy metabolism, appetite, as well as sympathetic nerve activity and immune response, potentially giving rise to interactive host-microbe signaling and gut-brain axis immune-inflammatory crosstalk (Xiong et al., 2004; Samuel et al., 2008).

Besides SCFA, gut microbiota-synthesized micronutrients such as vitamins exhibit beneficial value for both microbial and host metabolisms. Vitamin-K-producing gut bacteria namely *Bacteroides fragilis, Eubacterium lentum, Enterobacter agglomerans, Serratia marcescens*, and *Enterococcus faecium* (Fernandez and Collins, 1987; Cooke et al., 2006) anaerobically synthesize vitamin K2 (menaquinone) which is essential in decreasing vascular calcification, elevating HDL and lowering cholesterol levels, contributing to lower risk of cardiovascular disorders such as atherosclerosis and coronary heart disease (Kawashima et al., 1997; Geleijnse et al., 2004). Gut microbiota also serves as an important source of vitamins B for the host (Salminen et al., 1998; Degnan et al., 2014). Among them, vitamins B5 and B12, which are exclusively synthesized by intestinal microbiota, act as coenzyme for extensive range of host biochemical processes including production of acetylcholine and cortisol which are required for normal functioning of the nervous system. Deficiency of vitamins B5 and B12 have been linked to several disorders such as gastrointestinal discomfort, insomnia, neuropsychological and hematological disorders (Andres et al., 2004; Gominak, 2016). However, the possible link between loss of vitamin-producing gut microbiota and disease onset has not yet been elucidated.

Gut microbiota also plays an important role in the co-metabolism of bile acids with the host. These cholesterol derivatives are synthesized in the liver, followed by conjugation with taurine or glycine prior to storage in the gall bladder and subsequent secretion into duodenum to aid digestion, cholesterol and lipid metabolisms. In humans, 95% of bile acids are reabsorbed at distal ileum (Staels and Fonseca, 2009). The 5% unabsorbed primary bile acids are then bioconverted or deconjugated to secondary bile acids (predominantly DCA and LCA) by bile salt hydrolases secreted by several colonic microbiota such as *Clostridium perfringens* and *Clostridium scindens*, followed by partly colonic reabsorption and transportation back to liver for conjugation (Gopal-Srivastava and Hylemon, 1988; Ajouz et al., 2014) and the unabsorbed secondary bile acids are excreted by the host (Sorg and Sonenshein, 2008). Both primary and secondary bile acids are able to activate host nuclear FXR signaling, which in turn regulates the bile acids production, glucose metabolism, and potentially hepatic autophagy (Lee et al., 2014; Nie et al., 2015). Secondary bile acids serve as a potent activator of TGR5 (a member of the Rhodopsin-like subfamily of GPCRs) which is widely expressed on different tissues including in gall bladder, liver, spleen, intestine, and the immune cells. Such GPCR activation will stimulate second messenger c-AMP production, and the subsequent c-AMP-dependent downstream signaling, inducing expressions of a multitude of genes, whereby its physiological importance remains to be fully elucidated, pointing toward impacts of secondary bile acid-TCR5 axis (Hylemon et al., 2009). Secondary bile acids also possess antimicrobial effects which alter microbial cell membrane integrity, causing spillage of intracellular contents, thus inhibiting growth of bile acid-intolerant microbes (Nie et al., 2015). Such antimicrobial properties may contribute in shaping the composition of the gut microbiota and protecting the host from an array of infectious pathogens.

### Immunity and the Nervous System

Gut barrier which is composed of mucus layer and epithelial layer (containing several junctional protein structures that regulate barrier integrity and paracellular permeability), serves as the interface between the outside world and host internal environment. Disrupted gut barrier function will increase gut permeability to commensal microbes, microbial derived products (such as metabolites, virulence factors) as well as other luminal components, contributing to aberrant immune-inflammatory response such as inflammation, allergy, and autoimmune disorder mediated by molecular mimicry and dysregulated T-cell response (Barnaba and Sinigaglia, 1997). Such physical and immunological barrier’s function is cross-regulated by host-gut microbiota interactions. The regulation of gastrointestinal T-lymphocytes balance [regulatory T cell/T helper type 17 (Treg/T_H_17) ratio], which is crucial in maintaining intestinal homeostasis, discriminating between pathogens and commensal microbes via organizing “immune tolerance-productive immune response” status, is found to be involving the role of gut microbiota. Several commensal microbes such as *Bacteroides fragilis, Bifidobacterium infantis*, and *Firmicutes* are capable in inducing expansion of Treg cells such as FOXP3 expressing Treg and anti-inflammatory IL-10-producing Treg lymphocytes, which are crucial in suppressing pathological inflammation induced by aberrant effector T cells, hence fortifying gut barrier function (Paust et al., 2004; El Aidy et al., 2012; Lawley and Walker, 2013). Suppression of inflammatory response by Treg cells also play a critical role in inducing host tolerance in non-host cells, possibly enabling gut microbiota to construct their niches in host without being attacked by host immunity under normal circumstances. Furthermore, several microbiota-derived metabolites such as SCFAs had been shown to confer protection to the gastrointestinal barrier integrity against the disruptive effects of proinflammatory cytokines resulted by aberrant immune-inflammatory axis (Peng et al., 2007; Chen et al., 2017), whereby the mechanism remains unclear. On the other hand, commensal SFB was shown to induce intestinal T_H_17 cells and production of pro-inflammatory IL-17 and IL-22, enhancing antimicrobial defense and mucosal immunity in against of intestinal pathogen *Citrobacter rodentium* in gnotobiotic mice model (Ivanov et al., 2009). Besides, according to Atarashi et al. (2008), ATP which can be contributed by gut microbiota, is capable to activate differentiation of lamina propria cells (CD70 and CD11c) into T_H_17. In short, tight regulation of Treg/T_H_17 balance by healthy host-gut microbiota interactions are critical to prevent aberrant immune-inflammatory response.

Participation of gut microbiota in host immunity development and immune response regulation at both local and systemic levels had been demonstrated, whereby reversal of the differentiation-suppressed states of myeloid and lymphoid progenitor cells in GF mice was observed after colonization with normal microbiota, indicating gut microbiota facilitates maturation of haematopoiesis (innate immunity) and lymphocytopoiesis (adaptive immunity) (Khosravi et al., 2014). Intriguingly, a recent study by Erny et al. (2015) had revealed a novel function of host microbiota and their SCFAs in regulating maturation and functionality of microglia, the tissue macrophage of CNS. Colonization of GF mice with complex gut microbiota had shown partial reversal in the initial immature microglia phenotype in GF mice. Moreover, mice model with normal microbiota but deficit SCFAs receptor had mimicked the defective feature of microglia observed in GF mice, thus, indicating an important role of microbial-synthesized SCFAs in microglia homeostasis which is crucial in CNS health maintenance (Nishioku et al., 2010).

On the other hand, the emerging modulatory role of gut microbiota in the ENS had been documented in various studies. The ENS is crucial for life and is capable in autonomously regulating the physiology and function of the GIT, and bidirectionally communicate with the CNS via vagal pathways, forming the “gut-brain axis.” The major component of ENS is the EGC which resembles astrocyte in the CNS. EGCs form the enteric glial network that critically regulates a variety of GI functions including exocrine/endocrine secretions, gut motility, blood flow, and immune-inflammatory reactions, via a complex repertoire of calcium-dependent signaling (Ochoa-Cortes et al., 2015). Dysfunctions in ENS and EGC have been implicated in various gastrointestinal disorders (such as IBD, IBS, postoperative ileus), motility disorder (such as constipation), neurodegenerative disorder (such as PD) and infection-induced gut inflammation. Given the close proximity of gut microbiota and the ENS that located throughout the GIT, it is not surprised that gut microbiota can affect and modulate the development and function of ENS. In a study by Collins et al. (2014), region-specific significant reduction of myenteric nerve fiber density was observed in jejunum and ileum, but not duodenum of early postnatal GF mice compared to the SPF mice and ASF colonized mice. In addition, notably decrease in gut motility of GF mice compared with SPF and ASF counterparts was observed as well, reflecting the importance of gut microbiota in postnatal development of ENS in mid-to-distal small intestine, however, the reason underlies the region-specific myenteric ganglia reduction remains unclear. Another study by Kabouridis et al. (2015) had demonstrated marked reduction in mean number and density of mucosal EGCs in 8-week old GF mice compared to the conventionally-raised (CONV-R) counterparts, indicating the requirement of gut microbiota in normal development of mucosal enteric glial network. The same study also showed that gut microbiota-dependent EGC normal development does not restricted to critical early postnatal period as conventionalization of 4-week old GF mice demonstrated restoration of enteric glial network.

Toll like receptors (TLRs) from the microorganism-sensing PRR family serve a critical role in maintaining gut microbiota-host symbiotic relationship and intestinal homeostasis (Rakoff-Nahoum et al., 2004). Expression of TLR4 that recognizes the LPS of Gram-negative microbes, can be found on intestinal neurons and glial cells, possibly conferring ENS the ability to react straight to the stimulus derived from gut microbiota. The observation of decreased gut motility and lesser nNOS (neuronal NO synthase) neurons had been demonstrated in GF and antibiotic-treated mice, whereby such deficits are reproduced in TLR4-knockout mice as well as TLR signal transducer MyD88-knockout mice (Anitha et al., 2012), suggesting TLRs signaling might be the mediator between intestinal microbiota and ENS development. Furthermore, Soret et al. (2010) had reported a noteworthy elevation in ChAT but not nNOS-immunoreactive neuron, as well as increased cholinergic-mediated colonic circular muscle contractile reaction, induced by intracecal perfusion of butyrate, hence indicating potential regulatory effect of butyrate in ENS and gut motility.

### Colonization Resistance

Another important role of human microbiota is colonization resistance where indigenous microbiota confers protection to host against colonization of pathogenic invader and prevention against overgrowth of pathogenic microbiota members (pathobionts). Although the molecular basis of colonization resistance remains to be elucidated, the postulated mechanisms of actions can be classified into (1) direct interaction between human microbiota and pathogens in competing for shared niches and nutrients, and (2) exploitation or enhancement of host defense machinery by human microbiota to suppress pathogen (refer to **Table 2**).

**Table 2:** Colonization resistance mechanisms employed by gut microbiota.

Colonization resistance categories	Potential mechanisms involved in resisting colonization of pathogens	Reference
Direct interaction of gut microbiota with pathogenic invader or pathobiont	Niche exclusion: consumption on same limited resources to eventually out-compete and starve the competing pathogen. Alter ambient oxygen tension: suppress certain microbial virulence and survival Fermentation products (e.g., SCFAs): downregulate pathogens virulence factor; modulating intestinal pH to selectively inhibit microbial growth and promote growth of other microbes. Microbiota-host co-metabolite (e.g., secondary bile acids): antimicrobial property Antibiotic production: selective killing of microbes, modify microbiota composition Antibiotic detoxification (e.g., Beta-lactamase, efflux pump): microbial self-defense mechanism, removal of toxic molecules. Antimicrobial or toxin production (e.g., Bacteriocin): induce specific growth inhibition on members of the same or similar species.	Cherrington et al., 1991; Britton and Young, 2012; Kamada et al., 2013; McNally and Brown, 2015
Gut microbiota-mediated stimulation or enhancement of host defense mechanisms	Gut microbiota interacts with local pattern recognition receptors (PRRs) such as toll-like receptors (TLRs) and nod-like receptors (NLRs) signaling to facilitate maintenance of intestinal immunity homeostasis. Stimulate production of host antimicrobial peptides (e.g., Defensins). Induce secretion of immunoglobulin A (IgA), pro-inflammatory cytokines: recruitment of immune cells to eradicate pathogens. Induce activation of T helper type 17 (TH17) cells without intestinal pathology, enhancing resistance toward intestinal pathogen.	Ivanov et al., 2009; Lawley and Walker, 2013; Yiu et al., 2017

Dominant non-pathogenic gut microbiota members play a central role in occupying the niche and suppressing the growth and colonization of pathogens. However, during gut microbiome perturbation, decrease in dominant microbiota members reduces the colonization resistance capacity, allowing opportunistic pathogenic strains to invade or colonize the empty niches, leading to occurrence of infection. *C. difficile* is a classic example of pathobiont which we have discussed below.

## Gut Microbiome and Diseases

External factors (such as antibiotic consumption, dietary component, psychological and physical stress) and host factors can induce dysbiosis in gut microbiome. Dysbiosis is likely to impair the normal functioning of gut microbiota in maintaining host wellness, and potentially induce selective-enumeration of certain microbiota member including pathobionts, leading to dysregulated production of microbial-derived products or metabolites which might be harmful to the host, causing diverse range of diseases on local, systemic or remote organ (refer to **Table 3**), with some of the notable diseases, along with their respective microbiome-based therapy being discussed as below.

**Table 3:** Gut microbiome-associated human diseases and their respective dysbiotic features.

Disease categories	Specific diseases	Associated dysbiotic features	Reference
Immune-mediated/autoimmune diseases	inflammatory bowel disease (IBD)	Increase in virulent gut microbes (*Enterobacteriaceae* species, *Bacteroides fragilis*) and mucolytic *Ruminococcus* sp. Decrease in butyrate-producing *Firmicutes* (such as *Faecalibacterium prausnitzii, Roseburia hominis*)	Sokol et al., 2008; Png et al., 2010; Willing et al., 2010; Machiels et al., 2014
	Irritable bowel syndrome (IBS)	Increase in *Escherichia coli* Decrease in *Clostridium leptum* group of bacteria and *Bifidobacterium.* Decrease in bile acid biotransformation	Duboc et al., 2012
	Celiac disease	Increase in *Bacteroides–Prevotella* group Decrease in *Bifidobacterium* Varying observation (decrease or no change) in *Clostridium histolyticum, C. lituseburense*, and *Faecalibacterium prausnitzii* Alteration in SCFAs composition, but overall increase in total SCFA	Tjellström et al., 2005; Nadal et al., 2007; De Palma et al., 2010
	Systemic lupus erythematosus (SLE)	Increase in *Blautia* sp. and Gram-negative bacteria such as *Proteobacteria.* Decrease in gut microbiota diversity, *Odoribacter* sp., *Alistipes* sp. Increase in serum endotoxin	Shi et al., 2014; Luo et al., 2018
	Type-1 diabetes	Increase in *Bacteroidetes* Decrease in *Actinobacteria, Firmicutes*, and *Firmicutes/Bacteroidetes* ratio	Murri et al., 2013
	Rheumatoid arthritis (RA)	Increase in *Prevotella copri* and decrease in *Bacteroides* sp. In new-onset RA Increase in microbiota diversity of *Lactobacillus* genus in early RA	Liu et al., 2013; Scher et al., 2013
	Atopic disease (E.g., childhood allergic asthma)	Increase in fecal burden of *Clostridium difficile*, and *C. difficile/Bifidobacteria* ratio	Kalliomäki et al., 2001
Metabolic disorders/cardiovascular disorders	Obesity	Increase in *Firmicutes, Actinobacter* Varying observation (decrease, no change, increase) in *Bacteroidetes* Increase in glycoside hydrolase and SCFAs (butyrate and acetate)	Turnbaugh et al., 2006, 2009a; Koliada et al., 2017
	Type-2 diabetes	Increase in *Lactobacillus* Decrease in *Clostridium coccoides, Atopobium* cluster, and *Prevotella* Decrease in butyrate biosynthesis	Qin et al., 2012; Sato et al., 2014
	Hypertension	Increase in the Firmicutes/Bacteroidetes ratio, lactate-producer Decrease in microbiota diversity, acetate- and butyrate-producers	Yang et al., 2015
	Atherosclerosis	Increase in metabolites TMAO, endotoxin level (risk factor for early atherosclerosis)	Wiedermann et al., 1999; Koeth et al., 2013
Cancer	Colorectal cancer (CRC)	Increase in enterotoxigenic *Bacteroides fragilis*, and pathobionts *Fusobacterium* and *Campylobacter* sp. Decrease in butyrate-producer (*Faecalibacterium* and *Roseburia*)	Wang et al., 2012; Wu et al., 2013
Neurop sy chi atri c	Autism spectrum disorder (ASD)	Increase in *Clostridium* sp., *Bacteroidetes, Lactobacillus, Desulfovibrio* Decrease in *Bifidobacteria*	Song et al., 2004; Adams et al., 2011
	Alzheimer’s disease	Possible connection between gut microbiota-synthesized amyloids, LPS, γ-aminobutyric acid (GAB A – major inhibitory neurotransmitter), and the increased permeability of gut barrier and blood brain barrier with age	Pistollato et al., 2016
	Depression	Increase in genus *Eggerthella, Holdemania, Gelria, Turicibacter, Paraprevotella, Anaerofilum* Decrease in gut microbiota diversity, *Prevotella* and *Dialister*	Kelly et al., 2016
	Parkinson’s Disease	Increase in anti-inflammatory butyrate-producers from genus *Blautia, Coprococcus*, and *Roseburia* in patient fecal sample, pro-inflammatory *Proteobacteria* in patient mucosa Increased gene expression in LPS biosynthesis and microbial type III secretion system	Keshavarzian et al., 2015
Infectious disease	*Clostridium difficile* infection (CDI)	Increase in *Clostridium difficile;* Decrease in gut microbiota diversity and secondary bile acids-producing *Clostridium scindens*	Theriot et al., 2014
Uremic disease	Chronic kidney disease	Increase in *Firmicutes, Proteobacteria*, and *Actinobacteria* Decrease in *Lactobacilli*	Vaziri et al., 2013

### *Clostridium difficile* Infection (CDI)

*Clostridium difficile* is a Gram-positive toxin- and spore-producing anaerobe. It is also one of the *Firmicutes* members in normal gut microbiota. Catalytic activity of *C. difficile* toxins A (TcdA) and B (TcdB) damages cytoskeleton and colonic epithelial barrier integrity, thereby inducing aberrant inflammatory response and cell death (Genth et al., 2006; Pruitt et al., 2012). *C. difficile* infection (CDI)-associated symptoms include diarrhea, pseudo-membranous colitis, sepsis and death in severe cases (Bartlett, 1982). In America, around 453,000 incidences of CDI with 453,000 mortality cases were observed in 2011 alone (Lessa et al., 2015). Intriguingly, antibiotic administration was found to be the major risk factor for CDI (Pear et al., 1994). Around 5–35% antibiotic-treated individuals developed diarrhea as a common side effect. Association of *C. difficile* with antibiotic-associated diarrhea is the most frequent, which accounts for 10–20% of total incidences, compared to other pathogens such as *Staphylococcus aureus* and *Salmonella* species (Song et al., 2008). It was observed that greater incidence of diarrhea was correlated with uptake of antibiotics with broad-spectrum antibacterial effect (Bartlett, 2002). Also, *C. difficile* acquisition of resistant genes toward broad spectrum clindamycin, erythromycin, chloramphenicol, and linezolid mediated by multiple horizontal gene transfer modes (potentially via mobilizable transposon, phage transduction, conjugative plasmids) within *C. difficile* strains and possibly among commensal microbes had been reported (Johanesen et al., 2015). A cohort study by Pépin et al. (2005) discovered that broad-spectrum fluoroquinolone appeared to be the most potent risk contributor to *C. difficile*-associated diarrhea compared to other antibiotics. Precise mechanism of this antibiotic-associated diarrhea remains unknown, however, its noteworthy correlation with CDI inspires research on the relationship between gut microbiome and its pathogenic member – *C. difficile* in healthy non-disease state.

**FIGURE 1:**
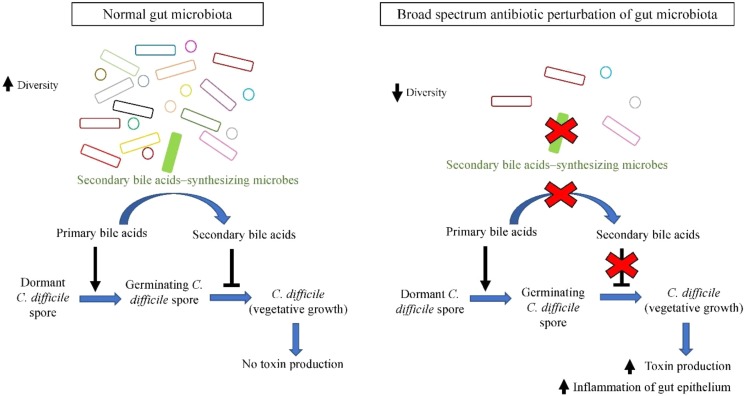
Secondary bile acids synthesized by members of gut microbiota (such as *C. scindens*) inhibit outgrowth of *C. difficile*, whilst broad spectrum antibiotic-induced dysbiosis favors *C. difficile* growth.

Currently it is postulated that dominant gut microbiota species confer protection to the host by employing colonization resistance mechanisms against overgrowth of *C. difficile* in normal microbiome. One of the proposed mechanisms is via the bio-conversion of primary bile acid to secondary bile acids. Primary bile acids (cholate derivatives) serves as germinant for *C. difficile* spores, whereas secondary bile acids (deoxycholate) inhibits vegetative growth of *C. difficile* (Sorg and Sonenshein, 2008). Antibiotic administration perturbs the gut microbial communities and reduces their diversity, especially secondary bile acids-synthesizing dominant microbes such as *C. scindens* (Antonopoulos et al., 2009). As a result, there is a significant reduction in microbial bioconversion of primary bile acids into antimicrobial secondary bile acids, leading to reduced inhibition of *C. difficile* vegetative growth, allowing *C difficile* outgrowth and colonization of the empty niches, leading to higher susceptibility of the host toward CDI (Samuel et al., 1973; Theriot et al., 2014), as illustrated in **Figure 1**. The increased toxin secretion by greater amount of vegetative *C. difficile* exerts greater damage on intestinal barrier, stimulating severe inflammatory response and causing impairment in intestinal ion absorption that leads to diarrhea.

Improved understanding on antibiotic-induced microbiome dysbiosis in pathogenesis of CDI and its recurrence has given rise to development of new promising therapeutic approach that involves restoration of gut microbiota, such as FMT. This therapy has successfully restored gut homeostasis via re-introduction of gut microbiota from healthy donor feces. Patients received FMT showed long-lasting elevation of fecal microbial diversity and a high recovery rate (90%) compared to vancomycin (narrow-spectrum antibiotic) therapy (recovery rate = 60%) (van Nood et al., 2013). Besides, elevation in gut *Bacteroidetes* and *Clostridium* cluster IV and XIVa (*Firmicutes*), and reduction in *Proteobacteria* were observed post-FMT (van Nood et al., 2013), indicating the importance of dominant microbes such as *Bacteroidetes* and non-pathogenic *Clostridia* member in suppressing *C. difficile* outgrowth. Similar study by Konturek et al. (2016) also had demonstrated a high healing rate (94%) of CDI post FMT treatment, with no recurrent CDI observed across 16 months follow-up. Besides, marked reduction of proinflammatory cytokines (such as TNF-α, IL-1β, IL-6, IL-8, and IL-12), significant elevation in plasma level of human antimicrobial peptide LL-37, along with increase in beneficial bacteria (such as *Lactobacillaceae, Ruminococcaceae, Desulfovibrionaceae, Sutterellaceae*, and *Porphyromonadaceae*) were observed in successfully treated patients post FMT. Although the exact beneficial strains and the underlying mechanisms of FMT in conferring high cure rate of CDI have not yet been clarified, these studies undoubtedly demonstrate a strong association between gut microbiome and development of CDI, and shed a light on the potential widespread use of microbiota displacement therapy in targeting CDI.

### Inflammatory Bowel Disease (IBD)

**FIGURE 2:**
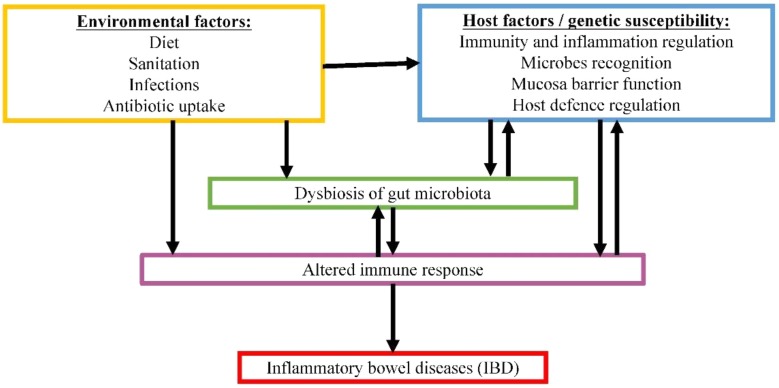
Proposed complex interactive relationships among gut microbiome, host factors and environmental factors in IBD pathogenesis.

Another example of gut microbiome-associated disease is IBD. IBD is a group of multifactorial, idiopathic, persistent and recurring gastrointestinal inflammations. Two common forms of IBD are CD and UC (Lennard-Jones, 1989). In CD, inflammation can occur anywhere along the whole GIT, whereas UC is only restricted to the large intestine. Both forms are associated with relapsing diarrhea, fever and abdominal pain. The occurrence of IBD is rising at alarming rate worldwide, estimating 1.4 million and 2.2 million individuals in America and Europe, respectively (Loftus, 2004). IBD generally involves host factors combined with environmental factors (Franceschi et al., 1987; Gent et al., 1994; Hugot et al., 2001). Although our understanding on the mechanism of pathogenesis for this disease is still lacking, crosstalk between gut microbiota and host factors show great potential in contributing to the disease development, as demonstrated in **Figure 2**. The inappropriate host immune response against gastrointestinal microbiota in genetically predisposed individual is speculated to be the main culprit in causing severe inflammation.

One of the possible etiologies for IBD is due to the hyper-responsiveness of T-lymphocyte toward non-pathogenic antigens presented on gut microbiota. Several studies have observed the developments of antibodies against commensal microbial antigens and autoantigens such as anti-*Saccharomyces cerevisiae*, anti-OmpC, perinuclear anti-neutrophil cytoplasmic antibody and anti-*Pseudomonas fluorescens*-associated sequence 12 (Duchmann et al., 1999; Landers et al., 2002). Also, it has been reported that each of these antibodies response patterns highly correlates with distinctive clinical characteristics, disease onset and severity, suggesting loss of different microbiota species that would affect the gut barrier function and gut immunity, results in different degree of gut inflammation (Vasiliauskas et al., 2000). In short, selective loss of tolerance toward gut microbiota in IBD patient with aberrant immune response leads to dysbiosis and loss of microbiota that might be responsible in maintaining the gut mucus barrier integrity. Impaired barrier function subsequently increases the exposure between gut microbiota and epithelial cells, causing further stimulation of local immunity, contributing to severe gut inflammation. All the studies mentioned above clearly suggest that dysbiosis in gastrointestinal microbiome might be a secondary consequence of gastrointestinal inflammation.

Conversely, Nagao-Kitamoto et al. (2016) suggested that gut dysbiosis potentially contributes to IBD pathogenesis. In this study, elevated pro-inflammatory gene expression was observed in GF mice colonized by gut microbiota isolated from IBD patients. Colitis-prone genetically predisposed GF mice colonized by IBD-associated-microbiota developed severe colitis compared to those that were colonized by healthy human microbiota. Together these findings strongly indicate bidirectional relationship between such disease and gut dysbiosis, in which dysbiosis potentially contributes to the onset of IBD and also serves as a secondary consequence of gut inflammation. Example of the dysbiotic features that are commonly observed in IBD patients is the reduction in gut *Firmicutes* such as *Faecalibacterium prausnitzii* and *Roseburia* sp. (Sokol et al., 2008; Willing et al., 2010; Machiels et al., 2014). These bacteria play an important anti-inflammatory role in reducing pro-inflammatory cytokines (IL-12, IFN-γ) and increasing anti-inflammatory IL-10 (Sokol et al., 2008). Besides, *Firmicutes* is important producer of butyrate, the primary energy substrates for colonocytes. Therefore, reduction in *Firmicutes* could elicit or heighten local inflammation by decreasing anti-inflammatory cytokine, an important regulator of mucosal immunity, and/or by SCFA-deficiency-induced impairment in colonic barrier function (Peng et al., 2007; Machiels et al., 2014). As such, it may be interesting to explore the therapeutic use of probiotic *F. prausnitzii* in managing IBD. Another dysbiotic feature observed in IBD patients is the elevation of virulent gut microbes such as *Enterobacteriaceae* species and *Bacteroides fragilis* with both having high endotoxic LPS in their outer membranes (Ruseler-van Embden and Both-Patoir, 1983; Darfeuille-Michaud et al., 1998). High endotoxic LPS expressed by microbiota has shown to induce gut inflammation and colitis development in mice, possibly via suppression of regulatory T-lymphocyte and/or activation of effector helper-T (T_H_-1/T_H_-17) through host TLR4 signaling pathway (Gronbach et al., 2014). Inversely, colonization of GF mice with low endotoxic microbiota prevented experimentally induced colitis (Gronbach et al., 2014), indicating that an increase in gut microbes with high endotoxic LPS and decrease in microbes with low endotoxic LPS in gut microbiome may lead to pathogenesis of IBD.

Another suggestion regarding the IBD-gut microbiome link is the initial impairment in gut mucus barrier function (either due to dysbiosis or other factors), resulting in elevation of mucus-eating (mucolytic) gut microbial species, which in turn aggravates the barrier function and stimulates severe inflammatory response. Gastrointestinal mucus layer and antimicrobial peptides (such as human defensins) secreted from epithelium work together as a barrier in preventing direct contact of luminal gut microbiota with gastrointestinal epithelial cells and inhibit aberrant inflammation (Johansson et al., 2008; Salzman et al., 2010). Colon biopsy samples of patients with UC showed reduced mucus layer, defective epithelium and deep microbe- and leukocyte-infiltration in colon mucosa (Swidsinski et al., 2007). Moreover, reduction in antimicrobial peptides such as Paneth cells alpha-defensins in patients with ileal CD was reported (Wehkamp et al., 2005). These findings propose that the impaired host antimicrobial defense in mucus barrier reduces the initial suppression of certain commensal microbiota such as mucolytic bacteria. This is further supported by Png et al. (2010) where they observed a significant elevation of mucolytic *Ruminococcus* sp. in gut microbiome of IBDs patients. Decrease in microbial growth inhibition by human defensins could enable outgrowth of mucolytic species, resulting in increased mucolytic species that feast on the mucus glycans, leading to deeper penetration of the inner mucosa layer and microbial infiltration, consequently, inflammation ensues.

Since IBD involves heightened host inflammatory response, current therapeutic approaches generally target the aberrant pro-inflammatory immune response at intestinal mucosa. Anti-inflammation therapies such as antibodies targeting proinflammatory cytokines (anti-TNF-alpha, anti-IL12, anti-IL23) and employing α4β7-integrin antagonist to inhibit trafficking of T-lymphocytes to gut tissue (Feagan et al., 2013; Christensen et al., 2015). However, IBD tends to recur in long-term, and chronic treatment with immuno-suppressive agents will develop neurotoxic side effects (Hyde et al., 1998; Croft et al., 2013). Therefore, development of a safer and more effective novel treatment is in great need. Given the association of IBD patients with decreased anti-inflammatory cytokines IL-10, administration of genetically modified probiotic (beneficial microorganism) such as *Lactococcus lactis* to express IL-10 had demonstrated a significant remission of disease activity in CD patients during trial (Braat et al., 2006). In this study, 80% CD patients demonstrated clinical improvements, with 50% showing complete remission, and no severe side effect was observed, thus, indicating a promising and safer maintenance treatment strategy for chronic intestinal disease, as compared to sole administration of inflammatory cytokines. However, 40% patients developed CD relapse upon discontinuation of such therapy, indicating the need of long-term prescription of this medication, which in turn will impact its economic viability. Besides, administration of prebiotics (fermentable polysaccharides) to stimulate growth and metabolic activity of beneficial protective gut microbes such as butyrate-synthesizing bacteria might be an attractive concept to treat gastrointestinal inflammation in IBD (Joossens et al., 2012; De Preter et al., 2013). Positive outcome had been reported in a randomized controlled trial by De Preter et al. (2013), whereby around 40–50% of CD patients with mild to moderately active disease achieved remission post treatment with prebiotic OF-IN. OF-IN intake induced a significant reduction in mucolytic *Ruminococcus gnavus* and a marked elevation of beneficial *Bifidobacterium longum*, which the latter was strongly correlated with the clinical improvement in CD disease activity (Joossens et al., 2012). This may due to the anti-oxidant and anti-inflammatory effects of *B. longum* in neutralizing reactive oxygen/nitrogen species (ROS and RNS) at site of inflammation, reducing gastrointestinal discomfort and tissue injury. Despite these exciting novel approaches in treating or managing IBD, the elusive etiology of IBD in each patient renders individual-specific variation in therapeutic response, posing a great challenge in designing a curative treatment that is effective to all.

### Celiac Disease

Furthermore, evidence has pointed to dysbiosis as the possible risk factor of autoimmune disorders, such as celiac disease. Other examples of gut microbiome-associated autoimmune disorders such as Type 1 diabetes, SLE and RA are depicted in **Table 3**. Celiac disease is a multifactorial chronic immune-mediated disorder in small intestine characterized by permanent intolerance to dietary gluten (such as gliadin peptides) and prolamines in genetically predisposed individuals expressing human leukocyte antigen (HLA)-DQ2 and/or HLA-DQ8 (Sanz et al., 2011). Celiac disease serves as one of the most prevalent lifelong disease in Europe, affecting 1% of the general population of all ages (Mustalahti et al., 2010), contributing to absolute mortality rate of 10.4 per 1000 person-years in celiac disease (Ludvigsson et al., 2009). In celiac patients, gluten triggers the activation of adaptive immune response in mucosa, inducing T_H_1 and T_H_17 mediated production of pro-inflammatory cytokines [for example IL-21, interferon (IFN)-gamma, TNF-alpha]; as well as innate immune response through stimulation of IL-15 synthesis, consequently leading to NKG2D-mediated enterocyte killing (Stepniak and Koning, 2006). Gliadin-mediated zonulin (a type of tight junction protein) signaling activation also had been reported, whereby it causes cytoskeleton rearrangement, reduced occludin – ZO-1 protein–protein interaction and increased gut permeability, allowing trafficking of luminal antigens to the submucosa (Drago et al., 2006). The etiology of celiac disease remains to be fully elucidated, with several factors including genetic predisposition, immunological factors (mucosal IgA), and environmental factors including gluten consumption, breast-feeding duration as well as gut infections (for example rotavirus infection), have been postulated to serve a concerted effort in contributing the risk and timing of celiac disease onset (Akobeng et al., 2006; Mulder and Mulder-Bos, 2006; Stene et al., 2006). These factors also play a critical role in shaping and altering gut microbiota composition, therefore potentially modulating gut microbiota roles in gut barrier function and immunity development.

Gut dysbiosis has been largely reported in celiac disease patients, in which the active phase of celiac disease is characterized by a remarkable decrease in Gram-positive bacteria, whereby this bacterial population is partly restored in patients that receive gluten-free diet, suggesting the initial reduction of the recovered species might be the secondary consequences of the disease. Besides, inconsistent observation encompasses of increase, decrease and indifferent abundance changes in commensal *Atopobium, Eubacterium rectale–Clostridium coccoides, Clostridium histolyticum, Clostridium lituseburense, Faecalibacterium prausnitzii, Escherichia coli, Lactobacillus-Enterococcus*, and *Staphylococcus* in celiac patients across different studies had been demonstrated (Nadal et al., 2007; De Palma et al., 2010; Marasco et al., 2016), suspecting existence of confounding factors (such as disease severity, disease duration, diets, age and sex-related factors of the experiment subject, and the experimental settings and sample types) which remain elusive. Despite that, a significant decrease in total Gram-positive to Gram-negative bacteria ratio was observed in all phases of celiac disease patients as compared to that of healthy controls, with marked reduction in “health-promoting” *Bifidobacteria*, and elevation in virulent Gram-negative *Bacteroides–Prevotella* groups being universally observed in multiple studies, reflecting possible pivotal role of *Bifidobacteria* and *Bacteroides–prevotella* in celiac disease (Nadal et al., 2007; De Palma et al., 2010; Marasco et al., 2016). Together, it is suggested that dysbiosis potentially plays both secondary and primary roles in celiac disease pathogenesis. Although such dysbiotic feature and its mechanistic link with celiac disease manifestation remain elusive, it is suspected that overgrowth of these pro-inflammatory endotoxin-secreting Gram-negative pathobionts may contribute to the gluten intolerance as well as the onset and/or progression of celiac disease, through the enhancement of the pro-inflammatory responses such as increased IFN-gamma and TNF-alpha production, which will impair gut barrier integrity and normal functioning. The resulting increased gut permeability could favor the infiltration of more luminal antigens (gluten and microbial products) to the submucosa, leading to amplification or perpetuation of inflammatory reactions. Intriguingly, despite the notable elevation in *Bacteroides–Prevotella* proportions, mucosal first line of defense mediated by secretory IgA was shown to be weaker in celiac disease patients, in which the IgA-coated *Bacteroides–Prevotella* was significantly lower than that of healthy control (De Palma et al., 2010). This finding postulates the possible defect in mucosal barrier of celiac disease patient which fails to stabilize gut microbiota, and loses its protective effect against invasion of noxious antigens and pathogens.

Another possible role of gut microbiota in contributing to celiac disease is their capability in synthesizing mTgs, which are the homologs of human-synthesized tissue transglutaminases (tTgs). The *mTg* gene, which is potentially associated with microbial survivability, is encoded in the genome of many commensal bacteria, with vast majority belongs to *Firmicutes* phylum. Transglutaminase, which is also the autoantigen in celiac disease, is a protein cross-linker in which it transamidates or deamidates gliadin peptides to form stable neo-compounds in a process called PTMP (Lerner et al., 2017a). The Tg-linked proteins are found to be autoimmunogenic in celiac disease patients, whereby induction of anti-tTG autoantibodies serves as a remarkable serological marker of celiac disease. Similar observation toward its microbial counterpart also had been documented, in which specific anti-mTg and mTg-gliadin neo complex antibodies were found in the serum of celiac disease patients but absent in healthy controls (Lerner and Matthias, 2015). Furthermore, mTgs which are extensively used as industrial food additives is capable in enhancing leakage of the epithelial tight junction, which is the major component of gut epithelial barrier in regulating the equilibrium between immune tolerance and immune response to non-self antigens. Tight junction dysfunction and “leaky gut” are commonly observed in autoimmune diseases including celiac disease, as the resulting increased gut permeability promotes entry of luminal immunogenic antigens, eliciting both local and systemic immune reactions, and the pathological autoimmune signaling cascade (Lerner and Matthias, 2015). Together, these findings speculate the endogenous mTgs synthesized by dysbiotic gut microbiota (especially dysregulation in *Firmicutes*/*Bacteroidetes* ratio) potentially abrogate the tight junction barrier integrity and drive autoimmunity through the intestinal luminal PTMP, in which the additional dietary source of mTgs may potentiate or exacerbate such deleterious effects.

Besides the aforementioned mTgs-mediated PTMP of dietary gluten by *Firmicutes*, PTMP-catalyzing ability had been reported in other commensal microbes, including those potentially virulent strains associated in the dysbiotic feature of celiac disease. *Bacteroides fragilis* of *Bacteroidetes* phylum was found to encode protein-modifier ubiquitin, suggesting potential aberrant ubiquitin especially in a dysbiotic configuration (such as over-representation of *Bacteroides* in celiac disease) might cross-react and interfere human ubiquitin activity, and its downstream signaling, as well as induce inappropriate activation of host immune (Patrick et al., 2011). Gram-negative bacteria, especially those pathogenic strains exhibit secretion systems to synthesize effector proteins with several enzymatic properties to manipulate host cellular signaling, for example via modification of host phosphoproteome to facilitate microbial survival and niche establishment in host (Grishin et al., 2015). Linking the dysbiotic features, microbial-mediated PTMP activities with the aberrant immune-inflammatory response in celiac disease, it is hypothesized that dysbiosis-mediated dysregulated PTMP activities causing both functional and conformational transformations of dietary and host proteins, generating novel aberrant epitopes which are potentially immunogenic and capable to interfere critical host cellular signaling, hence, triggering manifestation of autoimmune disease such as celiac disease (Lerner et al., 2016).

Alteration in SCFAs compositions and the SCFA total abundance in celiac children were also been documented by Tjellström et al. (2005). Significant increase in acetic, i-butyrate, i-valeric acid and the total SCFAs level were observed in celiac disease patients compared to that of healthy controls. Intriguingly, no significant difference of SCFAs composition and the total SCFAs was observed in the untreated celiac patient and gluten-free diet treated celiac patient, indicating the altered gut microbiota, particularly those SCFA-producing commensal microbes that responsible for such SCFAs composition profiles in celiac patients, might be long established in the genetic predisposed host before the onset of the disease, rather than a mere secondary consequence of celiac disease that can be corrected by eliminating gluten the “trigger” of celiac disease symptoms. However, the unknown bacterial origin in contributing such SCFA patterns in celiac patient renders a missing link between SCFA and the aforementioned key dysbiotic microbial communities.

Gluten-free diet has been largely employed to ameliorate the deleterious disease symptoms in celiac patient. However, gluten-free diet exhibits therapeutic limitation which it allows only a partial recovery of gut microbiota in celiac disease patients, both microbiologically (for example reduction in certain potentially pathogenic strains, incomplete restoration of beneficial *Bifidobacteria*) and metabolically (for example indifferent SCFA pattern in treated and non-treated patient), possibly due to the genetic factor of the patient, or the selective-enumeration of gut microbiota mediated by the absence of dietary gluten, which the underlying reason remains unclear. Strict adherence to total gluten-free diet appears challenging to the patient as well due to the vast application of gluten in processed food, hence alternative measures using microbiome-based therapy such as probiotic might be a promising option to manage celiac disease. Several *in vivo*, animal studies as well as human studies had demonstrated various beneficial effects of probiotics *Bifidobacterium* and *Lactobacillus* strains in ameliorating epithelial-gliadin induced deleterious effects. For example, *Bifidobacterium lactis* exerts inhibitory effect on the increased gut permeability induced by gliadin, and it protects the healthy curvy appearance of epithelial tight junctions from the unfavorable gliadin-induced “straightening” effect on tight junction in human colon Caco-2 cells (Lindfors et al., 2008). *B. longum* was shown to be capable in enhancing anti-inflammatory response by stimulating markedly high Treg cell mediated production of IL-10, suppressing pro-inflammatory T_H_1 cytokine IFN-gamma induced by fecal microbiota of celiac patient in a cell culture-based study (Medina et al., 2008). *B. longum* and *Lactobacillus casei* also have been reported to exert protective effect on animal model from gliadin-induced enteropathy, whereby *B. longum* can downregulate the production of pro-inflammatory TNF-alpha and reduce CD4+ T-cell mediated immune reactions (Laparra et al., 2012); whilst *L. casei* can induce complete recovery of gliadin-induced villus blunting, recover the basal TNF-alpha level, rescue homeostasis of GALT (gut-associated lymphoid tissue) (D’arienzo et al., 2011). Administration of probiotics *Bifidobacterium* in children with newly diagnosed celiac disease together with gluten-free diet had significantly reduced the potentially harmful *Bacteroides fragilis* abundance, decreased peripheral CD3+ T cells, and improved the disease symptoms (Olivares et al., 2014). Together, despite complete disease remission is not observed thus far, these studies demonstrate great therapeutic value of probiotics *Bifidobacterium* and *Lactobacillus* in ameliorating gliadin-induced toxic effects and improving celiac disease symptoms, indicating promising approaches in managing celiac disease.

### Obesity

In recent years, the newly identified factor – gut microbiome which is largely involved in host metabolism regulation, has been integrated into crosstalk studies between genetic factors, behavior and environmental factors as a possible contributor to obesity. Obesity is a global health hazard affecting more than 600 million people worldwide in 2014 (World Health Organization [WHO], 2016). It is associated with elevated energy intake and decreased energy expenditure, causing excessive fat accumulation with raised body mass index (BMI ≥ 30kg/m^2^), and is linked to metabolic syndrome, posing obese individuals to have a higher risk of developing obesity-associated disorders (for example cardiovascular disease, type-II diabetes, and liver abnormalities), low-grade inflammation, and premature mortality (Donnelly et al., 2005; Cani et al., 2007).

Metagenomics studies had discovered a significant increase in butyrate-producing *Firmicutes* and generally a decrease in *Bacteroidetes* were observed in distal colonic microbiome of obese patients and genetically obese mice, compared to their normal lean counterparts. These obesity-associated dysbiosis features were also accompanied by elevation in starch-degrading glycoside hydrolase and SCFAs (butyrate and acetate), and increased energy harvest capability as evidenced by markedly decreased fecal energy in obese mice (Ley et al., 2006; Turnbaugh et al., 2006), speculating significant elevation in host metabolism-related microbial communities would abnormally increase energy harvest, thus increasing the risk of developing obesity. This speculation is supported by gnotobiotic mouse model, whereby noteworthy elevations in body fat, fasting glucose and insulin levels, and development of insulin resistance that mimic physiological alterations in obesity patients, have been observed in GF mice post-colonization with a single saccharolytic gut bacterial species (*Bacteroides thetaiotaomicron*) and with gut microbiota from obese mice respectively (Backhed et al., 2004; Turnbaugh et al., 2006). Elevations in monosaccharides delivered to liver and liver triglycerides level due to enhanced starch metabolism facilitated by gut microbiota indicate a strong association between gut microbiota and host metabolism in glucose homeostasis and lipogenesis. These findings are consistent with previous studies where lipogenic substrates (such as monosaccharides and SCFAs) were able to trigger the expression of hepatic fatty acids-synthesizing enzymes like ACC-1 and FAS, promoting hepatic DNL (Higuchi et al., 2008). The subsequent storing of triacyl glycerides in adipocytes also has been shown to be possibly mediated by gut microbiota via suppressing of lipoprotein lipase-inhibitors (Backhed et al., 2004; Solinas et al., 2006). Together, this data suggests that over-representation of saccharolytic gut microbiota facilitates augmentation in food digestion, leading to higher energy harvest and increased fat deposition, thus contributing to obesity development.

Moreover, several studies suggest that increase in endotoxic LPS of Gram-negative gut bacteria contributes to obesity-associated metabolic syndrome. For instance, it was reported that elevated LPS led to obesity-associated insulin resistance and low-grade inflammation as demonstrated by Cani et al. (2007). Similarly, plasma LPS (metabolic endotoxemia) showed a marked increase in high-fat-fed mice, along with reduction of *Bifidobacteria*, a potential down-regulator of intestinal endotoxin (Griffiths et al., 2004). In investigation of the association between metabolic endotoxemia with obesity-associated metabolic disorders, continuous-LPS-infused mice were shown to develop fasting glycaemia, insulinemia, hepatic insulin resistance, increased hepatic and whole-body fat gains, similar to high-fat-fed mice phenotypes (Cani et al., 2007). In addition, absence of LPS-receptor resisted these adverse features regardless of LPS-infusion or high-fat diet treatments as shown in CD14 (LPS-receptor) knockout mice, indicating that the LPS/CD14 system mediates insulin insensitivity, thus inducing the onset of obesity and obesity-related metabolic disorders (Cani et al., 2007).

Since obesity-related metabolic disorder has been associated with gut microbiota dysbiosis, selective modulation of microbial composition via dietary intervention such as administration of prebiotics or probiotics might be a promising therapeutic approach. Administration of bacterial-synthesized CLA or CLA-producing bacteria such as *Lactobacillus rhamnosus* has shown a significant decrease in plasma cholesterol, triacyl glycerides and white adipose tissue in animal models (Koba et al., 2002; Lee H.Y. et al., 2006). Prebiotics such as inulin-type fructans that selectively nourish *Roseburia* and *Clostridium* cluster XIVa, and arabinoxylan that increases the abundance of *Bifidobacterium, Roseburia*, and *Bacteroides*, have demonstrated anti-adipogenic effect in high-fat-induced obese mice (Dewulf et al., 2011; Neyrinck et al., 2011). These findings demonstrate promising application of prebiotics and probiotics in obesity management, however, more supportive data from human models and clinical trials are required to justify these treatment strategies and their respective success rates.

### Colorectal Cancer (CRC)

Colorectal cancer (CRC) is the fourth leading cause of cancer-related mortality worldwide (Arnold et al., 2017). Similar to other cancer forms, CRC is a multifaceted diseases associated with genetic and environmental factors. Recent findings have suggested that gut microbiota plays a role in the intersection of these factors, probably through shaping a tumor-promoting environment. Colorectal carcinogenesis of the gut has been demonstrated by Li et al. (2012) using mouse model of APC-related CRC. In this study, GF mice displayed a significantly lower colonic tumor incidence and reduced tumor load as compared to that of conventionally raised mice, which the latter also exhibited other distinctive phenotypes of CRC such as rectal bleeding and anemia with a massive infiltration of inflammatory cells arising from a dysfunctional intestinal epithelial barrier at older ages, thereby suggesting gut microbiome and host factor (such as age and genetic predisposition) exert a concerted effort in contributing to CRC growth and progression. Conversely, tumor outgrowth into gut lumen would damage the intestinal barrier, resulting in increased infiltration of gut microbiota and potentially harmful luminal substrates into deeper tissue, leading to further stimulation of immune-inflammatory response and myeloid cell recruitment, which could in turn perturb the gut microbiome (Li et al., 2012). Together, gut microbiome and CRC display a bidirectional self-feeding relationship.

16S rRNA sequencing study of fecal microbiota of CRC patients has revealed a significant enrichment of *Bacteroides fragilis* (Wang et al., 2012; Wu et al., 2013). Despite the low detection limit of some metagenomics studies in providing strain-level information, it is suspected that possible bloom of enterotoxigenic strains of *B. fragilis* (ETBF) contributes to CRC through enhanced production of oncogenic *B. fragilis* enterotoxin (BFT). This speculation is consistent with the findings of Ulger Toprak et al. (2006) in which the *bft* gene expression was significantly higher in CRC patients than healthy controls. Wu et al. (2009) had demonstrated that colonization of tumor-prone mice with ETBF strains can trigger T_H_17-dependent colon tumorigenesis, possibly facilitated by its major virulence factor – BFT. BFT is cytopathic to intestinal epithelial cells and can cause colitis and colonic tumor, owing to its capability of catalyzing proteolytic degradation of tight-junction proteins ZO-1 and E-cadherin, leading to disruption of intestinal paracellular barriers (Moncrief et al., 1998; Wu et al., 1998). Moreover, BFT-mediated loss of membrane-associated E-cadherin could in turn activate T-cell factor–dependent Wnt (Wingless-Int)/β-catenin nuclear signaling in intestinal epithelial cells, stimulating oncogene *c-Myc* expression that results in persistent cellular proliferation (Wu et al., 2003). Furthermore, combined action of BFT and IL-17 on colonic epithelial cell can induce myeloid differentiation into pro-tumoral monocytic-myeloid-derived suppressor cells (MO-MDSCs), leading to selective upregulation of *Arg1* and NO synthase 2 (*Nos2*) thereby stimulating NO synthesis that could promote vascular endothelial growth factor (VEGF)-mediated tumor angiogenesis (Cianchi et al., 2003), and suppression of anti-tumor immunity (e.g., inhibit CD8+ T-cell proliferation) (Orberg et al., 2017).

On the other hand, non-colitogenic *Fusobacterium nucleatum* which is markedly enriched in CRC patients, is capable in inducing intestinal tumorigenesis in murine models, suggesting overrepresentation of the gut microbiota may be the driver for CRC that is non-associated with colitis or IBD (Castellarin et al., 2012; Wang et al., 2012; Wu et al., 2013). Although the causality and underlying mechanisms remain to be elucidated, it has been demonstrated that FadA adhesin of *F. nucleatum* could promote bacterial attachment and invasion into E-cadherin expressing CRC cells, subsequently activating β-catenin-regulated transcriptions, as shown by upregulation of lymphoid enhancer factor/T cell factor, host NF-κB, oncogene c-Myc and cyclin D1, consequently promoting CRC cell proliferation and inflammation (Rubinstein et al., 2013). Besides, *F. nucleatum* outer membrane protein, Fap2 also has been shown to mediate specific Fusobacterial attachment to Gal-GalNAc overexpressed in CRC, facilitating *F. nucleatum* invasion and colonization of CRC cells (Abed et al., 2016). In addition, involvement of TLR4/phosphorylated-p21-activated kinase 1/phosphorylated-β-catenin S675 cascade was found to be employed by invasive *F. nucleatum* as well (Wu et al., 2018). Briefly, both oncogenic ETBF and *F. nucleatum* along with their respective unique molecular signatures serve as potential biomarkers in identifying the risk of developing ETBF- and *F. nucleatum*-related CRC, and predicting malignancy progression. Antibiotics specifically targeting these pathobionts as well as the application of pharmacological inhibitors for pathogenic signaling pathways may be a promising approach to be ventured in. Further longitudinal studies are therefore recommended to assess the feasibility of such applications.

Microbial metabolites such as butyrate have been reported to inhibit proliferation and survival of tumor cells as evidenced by butyrate-induced upregulation of pro-apoptotic Bax, Fas, cell cycle checkpoint regulator p21 and p27, downregulation of anti-apoptotic Bcl-2, Bcl-xL, cyclin D1, and possible inhibition of LPS-induced activation of pro-inflammatory and pro-tumorigenic NF-κB signaling, via intracellular action of butyrate as HDAC inhibitor that could epigenetically regulate gene expression, and/or its extracellular action as a ligand that targets G-protein coupled receptor GPR109A-expressing cancer cell (Hassig et al., 1997; Thangaraju et al., 2009; Wei et al., 2016). Butyrate also exhibits chemoprotective effects in which colonocytes pre-treated with butyrate demonstrate higher resistance against hydrogen peroxide-induced oxidative stress and DNA damage, possibly via butyrate-mediated enhanced expression of GST M2 in colonocytes, which is a detoxifying isoenzyme specific for substrates that arise from oxidative stress, thereby reducing the impact of certain genotoxic CRC risk factors (Abrahamse et al., 1999; Ebert et al., 2003). Together with the observation of significantly reduced butyrate-producers *Faecalibacterium* and *Roseburia* in gut microbiota of CRC patients (Wang et al., 2012; Wu et al., 2013), it is speculated that the subsequent reduction in colonic butyrate could partly impair the anticancer immunosurveillance and potentiate tumorigenesis. Hence, the concept of increasing colonic butyrate either by supplementation of butyrogenic dietary fermentable carbohydrates (high fiber diet) or butyrate-producing probiotics exhibits great intervention potential on the prevention and therapy of CRC, whereby further studies are required to evaluate the therapeutic success rate, as well as to determine the effective concentration of butyrate *in vivo*, required to attain desirable clinical benefit. In a study by Femia et al. (2002), anti-tumorigenic activity of prebiotic OF-IN on azoxymethane-induced colon cancer rat model was demonstrated, as evidenced by significant reduction of tumor loads (adenoma and cancer) compared to control rat. However, serum metabolome such as butyrate level and the gut microbiota compositional change post-prebiotic treatment were not determined in this study. This makes it difficult to decipher which key microbiota and microbial products actually confer such anti-tumorigenic effect.

Accumulating data has also demonstrated protective effect of probiotics using lactic acid-producing bacteria (*Lactobacillus* and *Bifidobacterium*) against experimentally induced colon cancer, as shown by reduced tumor incidence and tumor loads. The underlying mechanisms are suggested to involve direct anti-proliferative effect on tumor cells, prevention of carcinogen-induced DNA damage, partly via inhibition of carcinogen and mutagen formation, reduction of pro-carcinogenic enzyme (ornithine decarboxylase) activity, and elevation of detoxifying enzyme activity, as well as enhancement of anti-tumor immunity as evidenced by increased natural killer cells, MHC class II antigen presenting cells, and CD4–CD8+ T cells post-probiotic treatment (Goldin et al., 1996; Pool-Zobel et al., 1996; Singh et al., 1997; Wollowski et al., 1999; Lee et al., 2004). Moreover, the gut microbiome is pivotal in influencing therapeutic efficacy of anti-cancer immunotherapy employing immune-checkpoint inhibitors. The anti-tumor effect of antibodies targeting CTLA-4, a major negative regulator of T-cell activation, is compromised in both GF mice and broad-spectrum antibiotics-treated mice, confirmed in both melanoma and colon cancer models, along with the observations of significantly reduced CD4+ T-cell and tumor-infiltrating lymphocytes. Such defects can be overcome by the administration of non-enterotoxin-producing strains of *B. fragilis*, immunization with immunostimulatory *B. fragilis* polysaccharide, or by adoptive transfer of *B. fragilis*-specific T-cells, in which the mechanisms underlie the restoration of therapeutic response to CTLA-4 blockade is suggested to be via induction of the IL-12-dependent T_H_1 immune response in tumor-draining lymph nodes, and stimulation of intratumoral dendritic cells (DCs) maturation (Vétizou et al., 2015). Similarly, Sivan et al. (2015) reported that *Bifidobacterium* could facilitate PD-L1 blockade efficacy via augmenting DCs function, enhancing CD8+ T-cell priming and accumulation at the tumor microenvironment level. The concept of combination treatment that encompasses anticancer immunotherapy and commensal microbes holds great promise for future CRC treatment.

### Autism Spectrum Disorder (ASD)

In recent years, increasing number of studies have explored a possible link between the gut microbiome and brain- or neuro-developmental disorders, such as ASD, with symptoms like mood disorder (depression), neurodegenerative Alzheimer’s and PDs (**Table 3**). ASD is a multifarious group of neurobiological disorders affecting 1 in 68, 8-year-old children in America (Christensen et al., 2016) and 1 in 160 children globally (World Health Organization [WHO], 2017). It is characterized by social and communication deficits, and repetitive, stereotyped behaviors (Constantino et al., 2000; Kim and Lord, 2012). Despite the poorly understood causative factor of ASD, several studies had observed a significant association between ASD, gastrointestinal dysfunction and dysbiosis in gut microbiota. Comorbidity analysis of ASD patients revealed high occurrence of gastrointestinal symptoms, with the severity positively associated with that of ASD (Wang L.W. et al., 2011). This co-occurrence is speculated to be implicated by impaired tyrosine kinase MET signaling which is important in brain development, gastrointestinal health and immune response regulation (Ieraci et al., 2002; Okunishi et al., 2005). This hypothesis is supported by the observation of impaired MET-signaling in ASD patients especially those that comorbid with familial gastrointestinal disorder (Campbell et al., 2009; Jackson et al., 2009). Also, clinical data has shown that physical health problems like chronic abdominal pain caused by gastrointestinal disorders aggravate self-injury and tantrum in ASD patients (Carr and Owen-Deschryver, 2007), thereby suggesting that treating the gastrointestinal dysfunction will possibly alleviate such ASD-associated symptoms.

During the investigation of microbiota composition in ASD patients, dysbiotic features such as increased *Clostridium* sp., *Bacteroidetes, Lactobacillus, Desulfovibrio*, and reduced *Bifidobacteria* were reported (Song et al., 2004; Adams et al., 2011), suggesting a possible link between dysbiosis and ASD. In a study by Shultz et al. (2015), mice treated with PPA via intra-cerebroventricular injection had shown to develop brain abnormalities and ASD-like symptoms. PPA is a type of SCFA, synthesized by enteric bacteria include the aforementioned *Clostridium, Bacteroidetes*, and *Desulfovibrio* species, but PPA level usually exist at low levels in normal healthy individuals. This suggests that possible bloom in PPA-producing gut microbes leads to elevated neurotoxic PPA concentration in blood, contributing to neuropsychiatric disorders. However, to date, dysbiosis-associated elevation in microbial-synthesized PPA in plasma of ASD patients has not been reported. Future studies in examining plasma PPA level in ASD patients are required. Surprisingly, treatment targeting gut microbiome has demonstrated beneficial effects in alleviating ASD-like symptoms in murine model. Desbonnet et al. (2010) reported that probiotic *Bifidobacterium infantis* improved the behavioral abnormalities and stress-associated gastrointestinal dysfunction which were initially induced in mice suffering MS. Reduction in *Bifidobacterium* is commonly observed in ASD patients, positive outcome post-probiotic treatment indicates a potential protective role of *Bifidobacterium* in neuro-immune aspects, suggesting probiotic *B. infantis* as a potential ASD therapy. Despite the unclear underlying mechanisms, antioxidant properties of *Bifidobacterium* are suggested to have a neutralizing neurotoxic effect, induced by excess NO in ASD (Yui et al., 2016). Further studies that mimic the human physiological environment are very much required to assess the therapeutic success and underlying mechanism of probiotic *B. infantis* in managing ASD.

## Perspectives and Future Direction

Emerging research on human microbiome and its role in human wellness and disease are currently making it to the limelight, by virtue of which, researchers of diverse clinical specialty areas are attempting to fit this potential “missing piece of puzzle” into existing disease models, particularly those with unknown etiology. Tremendous data has demonstrated strong association of the gut microbiome with host metabolism, immune and neuroendocrine homeostasis, and the possible dysregulation or alteration of gut microbiome. Perturbation of such delicate equilibrium will in turn contribute to disease manifestation. However, the causal or correlation link remains debatable due to lack of direct evidence and mechanistic details. Our understanding on gut microbiome is still at a very preliminary stage, whereby there are several limitations and research gaps that are worth further exploration to better justify the link. For instance, large-scale longitudinal studies that account for subject-specific variations in characterizing the human microbiome are strongly required. On the other hand, another important component of human microbiome – the human virome that is dominated by bacteriophage (viruses that infect bacteria), is relatively less established compared to that of commensal bacteriome research. Only recently the presence of bacteriophage (or phage) in the human microbiome has been highlighted. Their potential role in modulating immune homeostasis has been suggested via (1) indirect mechanisms such as through regulating bacterial microbiome composition (phage-mediated lytic killing of host bacteria) and function (phage-mediated introduction of novel functional genes to lysogenic host such as production of endotoxin and antibiotic resistance), and (2) direct interference with human immune-inflammatory responses such as downregulation of the pro-inflammatory NF-κB pathway, inhibition of excessive ROS synthesis, induction of anti-inflammatory IL-10 production, and T4 phage adhesin gp12 - mediated counteraction to LPS-induced inflammation, as evidenced by downregulation of inflammation markers IL-1α and IL-6, and diminished leukocyte infiltration (Górski et al., 2016; Miernikiewicz et al., 2016). In addition, simultaneous increase in pro-inflammatory markers such as IL-1β, IL-1α, along with upregulation in anti-inflammatory IL-1 receptor antagonist and SOCS3 have been observed in *P. aeruginosa* and *S. aureus* phages treated cells, and the authors postulate that at least some phages have evolved anti-inflammatory properties to aid their survival and propagation, while retaining some pro-inflammatory properties (Van Belleghem et al., 2017). On the contrary to the largely reported anti-inflammatory properties of phage, significantly increased abundance of bacteriophages and changes in phage communities composition in CD patients compared to healthy controls have indicated a possible contributory role of phage in aberrant immune response (Lepage et al., 2008; Wagner et al., 2013). Undeniably, integrating human virome studies into the existing human bacterial microbiome will provide a bigger and clearer picture on how such transkingdom interactions could impact human health, thus, facilitating disease etiology studies. However, overcoming the obstacles such as limitations in virome sequencing techniques, uninformative database for characterization of novel viral genomes and the difficulties in identifying origin of virus from patient sample followed by deciphering the underlying mechanisms are urgently required.

Another noteworthy aspect is that the observed association of human microbiome with the host in healthy and disease states in fact reflects a far more complicated interactive relationship, rather than a mere unidirectional “cause and effect.” Hence, experimental design should be revised and tailored accordingly to comprehensively investigate at which point of the disease (for example: disease onset, early disease stage, disease progression, active or latent disease) does the human microbiota play a role in, as this will reveal either the primary role, secondary role, or both roles undertaken by human microbiota in disease manifestation. Besides, varying observations such as dysbiotic features in specific diseases, and host response toward introduced stimuli across published literatures on this topic, suspecting the existence of confounding factors, for example, subject-specific differences or distinctive experimental designs across studies becomes important. Subject-specific factors such as genetic, age, gender, lifestyle, diet, infection, disease and postnatal exposure to maternal and environmental microflora serve a crucial role in shaping the unique composition of human microbiota in each individual. Also the commensal microbial composition will in turn affect the development, maturation and normal functioning of the host factors (such as the neuro-immune network development and maturation), will contribute toward issues on varying disease susceptibility. Interconnection of these factors make it much more challenging to design constructive and conclusive experiments to test the role of the microbiome in targeted disease and establishing the true human microbiome-host relationship in multifactorial disease conditions. Whilst for the experimental design, differences across studies, standardization of research protocols are required to enable effective comparison of findings across studies, reduce data variance and biases, and producing sound and robust findings for better explanation of human microbiome-disease relationships.

A better understanding of human microbiome and how the commensal microbes interact with the host is undeniably useful to delineate the etiology and pathophysiological aspects of several human diseases, as well as developing a more effective therapeutic option to counteract the limitation of currently existing treatments. Application of FMT, probiotic and prebiotics derived from advances in gut microbiome studies to ‘rectify’ or ‘restore’ the altered gut microbiota in the dysbiosis-associated disease state back to the ‘healthy’ equilibrium, holds great promise as the alternative therapeutic option in several symptomatic disease management. However, the beneficial therapeutic effect of microbiome-based therapy is largely dependent on the role of dysbiosis in contributing to the nature of the disease. Accurate identification of key microbiota members, intricate selection of microbial strains used in probiotics, or types of prebiotics administered to selectively enumerate the desirable commensal, have definitely added extra challenges to the extensive application of microbiome-based therapy to future clinical practices. In addition, majority of the probiotics are generally regarded as safe but it does not warrant permanent safety. Several potential risks include possible transfer of antibiotic resistance gene or virulent genes among microbes. Although severe systemic side effects post-microbiome therapy are not observed to date, minor gastrointestinal discomforts such as abdominal pain, vomiting, nausea are commonly reported. This subsequently leads to withdrawal or non-compliance by patients during field trials. Therefore, improvement on treatment regime, route of administration, and effective communication with the patients are strongly encouraged. In short, Human Microbiome field of research is still relatively new but rapidly growing, showing several preliminary but promising studies on the modulatory role of human microbiome in human wellness and disease. Future applications of microbiome-based disease diagnosis, prognosis monitoring, prophylaxis and treatments which exhibit great potential in revolutionizing the current measures in disease management and treatment, are definitely worth anticipating.

## Author Contributions

ZK conducted the literature search and compiled the information. SL contributed academic assistance and edited the manuscript.

## Conflict of Interest Statement

The authors declare that the research was conducted in the absence of any commercial or financial relationships that could be construed as a potential conflict of interest.

## Abbreviations

ACC-1: acetyl-CoA carboxylase
APC: adenomatous polyposis coli
Arg1: arginase 1
ASD: autism spectrum disorder
ASF: altered Schaedler flora
ATP: adenosine triphosphate
BFT: *Bacteroides fragilis* enterotoxin
BMI: body mass index
c-AMP: cyclic adenosine monophosphate
CD: Crohn’s disease
CD14: cluster of differentiation 14
CDI: *Clostridium difficile* infection
chAT: choline acetyltransferase
CLA: conjugated linoleic acid
CNS: central nervous system
CoA: coenzyme A
CRC: colorectal cancer
CTLA-4: cytotoxic T lymphocyte protein 4
DCA: deoxycholic acid
DNL: *de novo* lipogenesis
EGC: enteric glial cell
ENS: enteric nervous system
ETBF: enterotoxigenic *Bacteroides fragilis*
FAS: fatty acid synthase
Fiaf: fasting-induced adipose factor
FMT: fecal microbiota transplant
FOXP3: forkhead box P3
FXR: farnesoid X receptor
GABA: γ-aminobutyric acid
GF: germ free
GIT: gastrointestinal tract
GLP: glucagon-like peptide
GPCR: G protein-coupled receptor
GST: glutathione *S*-transferase
HDAC: histone deacetylase
HDL: high-density lipoprotein
IBD: inflammatory bowel disease
IBS: irritable bowel syndrome
IFN-γ: interferon gamma
IgA: immunoglobulin A
IL: interleukin
IPA: indole-3-propionic acid
LCA: lithocholic acid
LPS: lipopolysaccharide
MET: mesenchymal epithelial transition
MHC: major histocompatibility complex
MS: maternal separation
mTg: microbial transglutaminase
NF-κB: nuclear factor-kappa B
NKG2D: natural-killer group 2 member D
NLR: nod-like receptor
NO: nitric oxide
NOS: nitric oxide synthase
OF-IN: oligofructose-enriched inulin
OmpC: *Escherichia coli* outer membrane protein
PD: Parkinson’s disease
PD-L1: programmed cell death protein 1 ligand 1
PPA: propionic acid
PRR: pattern recognition receptor
PTMP: post-translational modification of proteins
PXR: pregnane X receptor
RA: rheumatoid arthritis
RNA: ribonucleic acid
RNS: reactive nitrogen species
ROS: reactive oxygen species
SCFA: short-chain fatty acid
SFB: segmented filamentous bacteria
SLE: systemic lupus erythematosus
SOCS3: suppressor of cytokine signaling 3
sp: species
SPF: specific-pathogen free
TcdA: *Clostridium difficile* toxin A
TcdB: *Clostridium difficile* toxin B
Tg: transglutaminase
T_H_: helper T-cell
TLR: toll-like receptor
TMAO: trimethylamine N-oxide
TNF: tumor necrosis factor
Treg: regulatory T cell
tTg: tissue transglutaminase
UC: ulcerative colitis
WGS: whole genome sequencing
WHO: World Health Organization
Wnt: Wingless Int
ZO-1: zonula occludens-1
